# Antidromic Atrioventricular Reentrant Tachycardia Dependent on a Unidirectional Left Anterior Accessory Pathway Mimicking Peri-mitral Ventricular Tachycardia: Successful Ablation via a Transseptal Approach

**DOI:** 10.7759/cureus.3060

**Published:** 2018-07-27

**Authors:** Aamir H Khan, Maryam H Khan, Hunaina Shahab, Qamaruddin Roziman

**Affiliations:** 1 Medicine, Aga Khan University Hospital, Karachi., Karachi, PAK; 2 Medicine, Ziauddin Medical College, Ziauddin University, Karachi, PAK; 3 Medicine, Aga Khan University Hospital, Karachi, PAK

**Keywords:** ventricular tachycardia, supraventricular tachycardia, ablation, transseptal approach, antidromic atrioventricular reentrant tachycardia

## Abstract

Antidromic atrioventricular reentrant tachycardia (aAVRT) is rare compared to orthodromic atrioventricular reentrant tachycardia (oAVRT). An aAVRT that is dependent on a unidirectional, decremental accessory pathway (AP) is even rarer. Idiopathic ventricular tachycardias (iVT) that have benign prognoses and respond well to medical therapy can be confused with aAVRTs dependent on APs having ventricular insertion sites close to the iVT focus and have a real risk of sudden death. The preferred approach of ablation for such tachycardias with anterograde conduction only is a retrograde aortic approach, which facilitates the mapping of the earliest ventricular activation during atrial pacing or tachycardia from the ventricular side. This, however, necessitates access to the arterial system with accompanying complications. We describe herein the case of a wide complex tachycardia, which was treated initially as VT with intravenous lidocaine. The baseline electrocardiogram (ECG) did not show preexcitation. An electrophysiology study (EPS) revealed a left anterior AP that conducted anterograde only. AVRT was easily inducible at a cycle length of 290 ms. Successful ablation was undertaken via the transseptal approach without recurrence.

## Introduction

Antidromic atrioventricular reentrant tachycardia (aAVRT) is rare compared to orthodromic atrioventricular reentrant tachycardia (oAVRT) [1]. Accessory pathways (AP) causing aAVRT, which have a unidirectional, decremental conduction, have been sporadically reported. Cases of left-sided anterograde decremental pathways, including one left posterior, one left posteroseptal, three left free-wall, four left nodo-fascicular or nodo-ventricular pathways and one on the supero-septal aspect of the mitral annulus have been reported in the past [2]. We describe the case of a young man presenting with a first episode of tachycardia mimicking a peri-mitral ventricular tachycardia (VT), which was successfully ablated via the transseptal approach.

## Case presentation

**Figure 1:**
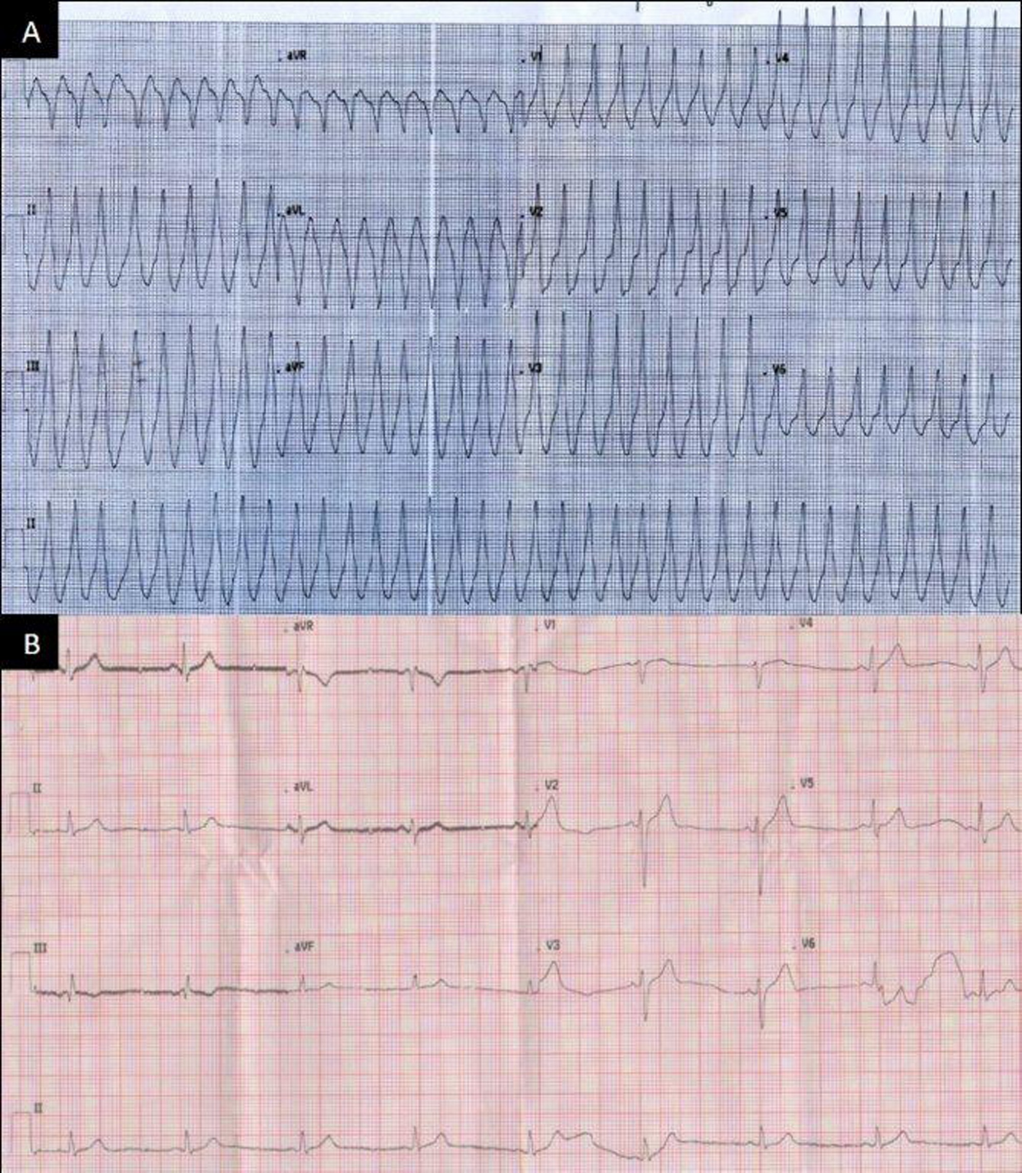
Electrocardiogram A: ECG at presentation showing a wide complex regular tachycardia at 214 bpm, mimicking a ventricular tachycardia with RBBB morphology, positive concordance, axis +120, R in lead V5. Likely focus anterolateral peri-mitral region. B: Baseline ECG after termination of the wide complex tachycardia. There is no evidence of preexcitation or signs of myocardial ischemia. ECG: electrocardiogram; RBBB: right bundle branch block

A 37-year-old man without any prior comorbid conditions presented to a secondary-care hospital with a first episode of a wide complex tachycardia of two hours duration (Figure 1A). Besides chest discomfort and thumping sensation, no other symptoms were noted. He was treated for VT in the emergency room. A single 1 mg/kg dose of lidocaine terminated the tachycardia. Subsequently, a 12-lead electrocardiogram (ECG) pattern was obtained essentially within the normal limits with no suggestion of preexcitation or ischemia (Figure 1B).

He was then referred to our center for further evaluation and underwent further testing with a normal ECG and normal serial cardiac enzymes. His coronary angiogram revealed normal coronary arteries. In view of his ECG, the differential diagnosis included a VT arising from the anterolateral peri-mitral area and an aAVRT arising from an AP in the left free wall. He then underwent an electrophysiologic study (EPS) to rule out an AP.

**Figure 2:**
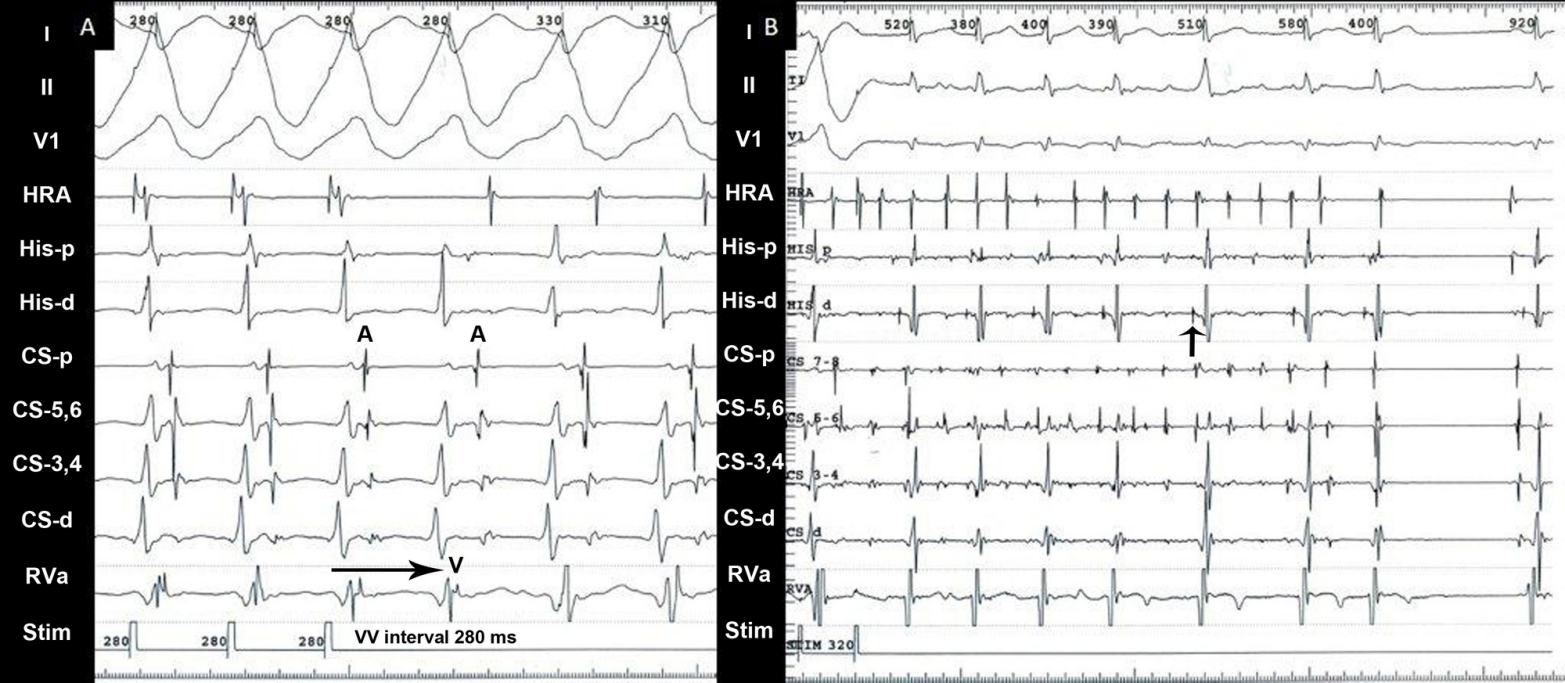
Electrogram A: The tachycardia was entrained from the HRA at 280 ms without a change in the QRS morphology. The tachycardia cycle length was 310 m. An AVA response is noted at cessation of entrainment. This differentiates aAVRT from VT where an AVVA response is seen. B: Atrial fibrillation induced during pacing from the HRA. Pre-excitation is noted during pacing but conduction over the AP is noted to relatively slow down or be blocked during atrial fibrillation. This is reflected by His deflections seen prior to each conducted complex, signifying impulse conduction primarily down the AVN. This can be explained by an ERP of the AP equal to or more than AVN ERP or decremental AP conduction due to increased atrial rate. AVA: atrium-ventricle-atrium; aAVRT: antidromic atrioventricular reentrant tachycardia; VT: ventricular tachycardia; AVVA: atrium-ventricle-ventricle-atrium; HRA: high right atrium; AVN: atrioventricular node; ERP: effective refractory period; AP: accessory pathway; His - p: His proximal; His - d: His distal; CS: coronary sinus; RVa: right ventricle apex; Stim: stimulation channel; A: atrial electrogram; V: ventricle electrogram; VV interval: ventricular cycle length

After femoral venous access was achieved, two quadripolar catheters were placed in the high right atrium (HRA) and right ventricle (RV). A decapolar catheter was placed in the coronary sinus (CS) from the femoral vein but could not be advanced distally enough to bracket the AP, due to the CS anatomy. The ablation catheter was positioned at the His bundle region. EPS was carried out in the usual manner. Baseline intervals were within the normal limits. Retrograde conduction with ventricular pacing was concentric and decremental. Atrial pacing revealed preexcitation at 360–380 ms initially and 290–310 ms later; this occurred briefly during Wenckebach block in the atrioventricular node (AVN) with induction of tachycardia. Effective refractory periods (ERP) of anterograde AVN and APs were <220 ms. Tachycardia was induced during atrial and ventricular pacing by atrial and ventricular extrastimulation. Tachycardia cycle length (TCL) varied from 440 to 280 ms (after warm-up) in the initial and later phases of the study. The earliest local ventricular activation during tachycardia was at the distal CS, suggesting either a VT with 1:1 retrograde atrial activation or an aAVRT involving a left free-wall AP. The tachycardia was entrained from the HRA, giving an atrium-ventricle-atrium (AVA) response (Figure 2A) to the cessation of entrainment, which differentiated the aAVRT from VT [3]. Atrial fibrillation was induced during atrial pacing and terminated spontaneously, but it did not show gross preexcitation as was noted during the tachycardia (Figure 2B).

**Figure 3:**
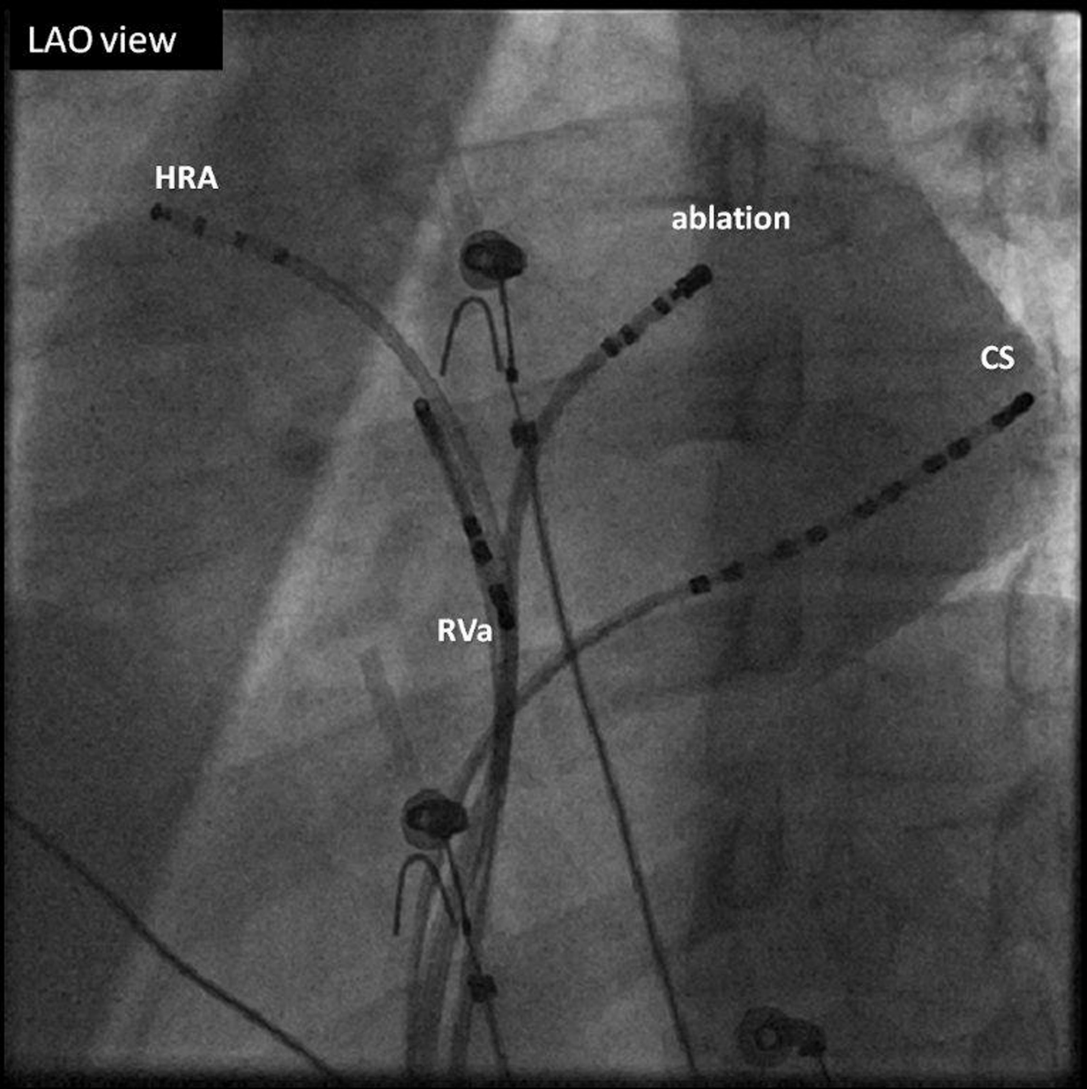
Fluoroscopic Image LAO 37 degrees projection: The catheters are noted in the HRA, RV, and CS and the ablation catheter in the LA through a transseptal puncture. The ablation catheter is at the ablation site (12 o'clock at the mitral annulus). LAO: left anterior oblique; HRA: high right atrium; RV: right ventricle; CS: coronary sinus; LA: left atrium His catheter is the ablation catheter.

A transseptal puncture was then carried out using an 8 Fr Mullins sheath. Heparin 5000 units were given intravenously after attaining left ventricle (LV) electrogram. Subsequently, heparin was administered intravenously to maintain the activated clotting time (ACT) at >250 s. Mitral annulus was mapped in the left anterior oblique (LAO) at about 30 degrees during tachycardia, as the atrial pacing cycle length (CL) that caused preexcitation would invariably induce tachycardia. At the 12 o'clock position (Figure 3), the earliest ventricular signal was noted.

**Figure 4:**
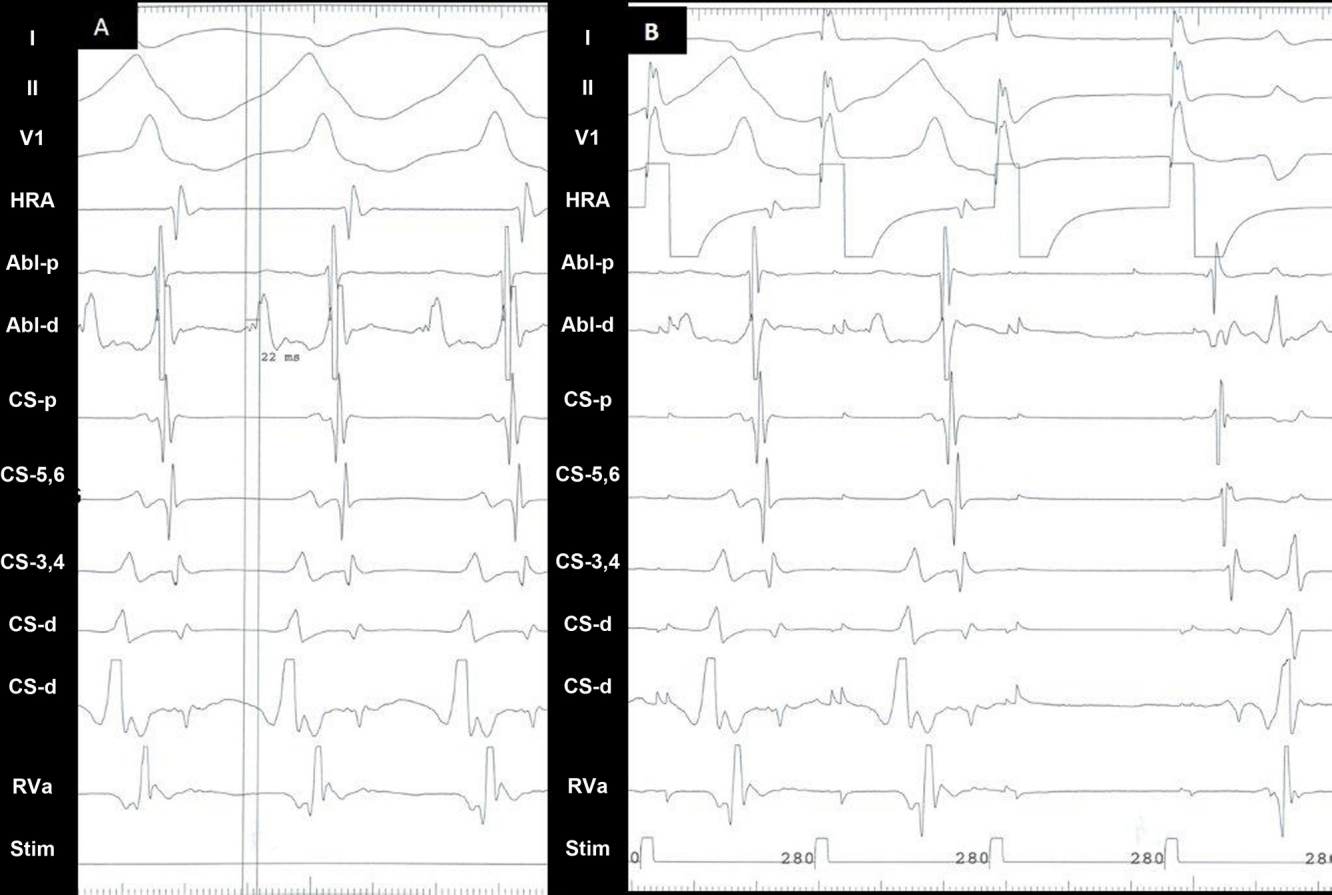
Electrogram A: Mapping during tachycardia shows the ventricular electrogram at the ablation catheter to be earlier than the surface QRS by 22 ms at the ablation site. B: Pacing was started at 280 ms during the tachycardia just prior to the application of radiofrequency energy in order to ensure the stability of the catheter with the termination of tachycardia and resumption of normal sinus rhythm. Application of radiofrequency energy terminates the tachycardia with a block in the accessory pathway. Last noted electrogram is atrial. Post-termination pacing continues. HRA: high right atrium; Abl - p: ablation catheter proximal; Abl - d: ablation catheter distal; CS: coronary sinus; RVa: right ventricle apex; Stim: stimulation channel

The fractionated component of the ventricular signal was measured 22 ms prior to the surface delta wave (Figure 4A). To ensure catheter stability, atrial pacing was continued at the TCL (280 ms). Radiofrequency (RF) was delivered at the spot at a temperature of 600 degree Celsius and at 50 Watt. Tachycardia terminated in the AP (Figure 4B) within 7 s of RF application, which was continued for 62 s. Thereafter, no tachycardia was inducible. A post-ablation AVN Wenckebach block occurred at 290 ms without the induction of tachycardia or preexcitation. Post-ablation right ventricular apical and basal pacing revealed nodal conduction.

## Discussion

Although very rarely iVT can have a malignant potential, classically they have been described as benign [4]. aAVRT, on the other hand, has a finite sudden cardiac death risk of 0.02%/patient/year [5]. aAVRT of the right side has been more commonly described. Left-sided APs that conduct anterograde only and have decremental properties have been rarely described. Of these left APs, the left anterior position (12 o'clock on the mitral valve) is even rarer. There is evidence that these unidirectionally conducting left APs behave more like the classical Mahaim pathways with unidirectional and decremental conduction [2].

In our case, the AP demonstrated unusual characteristics: it had no retrograde conduction and was slowly conducting initially, with conduction mainly apparent in the anterograde direction in tachycardia; there was no preexcitation at the baseline, which can be explained by the distance between the AP and the sinus node. Pacing from the distal CS did not circumvent this issue due to the distance between the CS pacing site and the anterior position of the AP. After induction of atrial fibrillation, the preexcitation present during atrial pacing attenuated, which may be consistent with the decremental properties of the AP.

In the literature, the acceptable method of ablation of such APs is a retrograde aortic approach. APs have a 1–3-mm atrial insertion, but at the ventricular end, these APs ramify and travel some distance into the ventricle toward the apex [6]. Most of these rare cases have been ablated from the subannular (retroaortic approach); wherever the transseptal approach was tried, recovery of the AP was immediate, necessitating the change of strategy [3].

In our case, we mapped the mitral annulus for the earliest ventricular signal from the left atrial side. We tracked the ventricular intracardiac electrogram to the earliest signal. A fractionated component was noted at the 12 o'clock site (Accessory pathway potential), which was well before the surface delta wave. RF application terminated the aAVRT in the AP (termination on atrial signal) within 7 s. 

## Conclusions

Our case demonstrates that unidirectional, anterograde conducting APs can be successfully ablated via the transseptal approach obviating the need for access to the aorta with its incumbent risks. The transseptal approach also allows for better maneuverability in the anterolateral and lateral positions.
